# Association between HIC1 promoter methylation and solid tumor: A meta-analysis 

**DOI:** 10.17179/excli2020-1102

**Published:** 2020-04-07

**Authors:** Tie Zhao, Justice Afrifa, Dong Wang, Jingcui Yu

**Affiliations:** 1 Scientific Research Centre, The Second Affiliated Hospital of Harbin Medical University, Harbin 150081, China; 2 Department of Medical Laboratory Science, University of Cape Coast, Cape Coast, Ghana

**Keywords:** HIC1, hypermethylation, tumor suppressor gene

## Abstract

The epigenetic silencing of tumor suppressor genes by promoter methylation plays an increasingly important role in cancer research. A number of studies have reported the contribution of HIC1 promoter methylation towards the occurrence and development of solid tumors, even though HIC1 promoter methylation has also been found in normal and benign tissue samples. We sought to perform a more accurate and comprehensive meta-analysis to assess the association between HIC1 promoter methylation and cancer risk. We searched and retrieved all published studies on HIC1 promoter methylation in PubMed, Google Scholar, Embase, Cochrane Library, and Web of Science databases. After two reviewers checked the studies and extracted the necessary data independently, the meta-analysis was performed using STATA 12.0 software. A total of 14 case-control studies (949 cancer patients, 282 benign, and 371 normal controls) were included in our study. We report a significantly elevated HIC1 promoter methylation in tumor samples compared to normal (*OR* = 7.02, 95 % *CI* 3.12-15.78, *P* < 0.001) and benign controls (*OR* = 2.69, 95 % *CI *1.13-6.42, *P* = 0.025). Subgroup analysis stratified by ethnicity showed a significantly reduced heterogeneity among North American *(I^2^* = 0.0 %, *P *= 0.502) and European (*I^2^* = 33.7 %,* P* = 0.183) samples. In addition, heterogeneity was significantly reduced among MSP based detection method (*I^2^* = 36.4 %, *P* = 0.139) when samples were stratified based on the methylation detection methods. The overall outcome demonstrated that HIC1 promoter methylation may be involved in the occurrence and development of solid tumors and has the potential to serve as an epigenetic maker in various specific tumors.

## Introduction

Cancer is a leading cause of death in both developed and developing countries. The burden is expected to grow worldwide due to the growth and aging of the population (Torre et al., 2015[29]). For a long time, cancer has been considered as an event caused by external environmental and genetic modifications including point mutations, amplification in oncogenes, and absence in tumor suppressor genes. However, in the last few decades, it has become increasingly clear that altered epigenetic regulation plays a key role in many different diseases, particularly cancers (Ziogas and Roukos, 2009[40]). 

Alterations of DNA methylation have been recognized as an important component of cancer development. Hypomethylation, in general, arises earlier and is linked to chromosomal instability and loss of imprinting, whereas hypermethylation is associated with promoters and can arise secondary to gene silencing and thus might be a target for epigenetic therapy (Daura-Oller et al., 2009[9]). Over two decades ago, after observing the hypermethylation of this specific gene in breast, colon, fibroblast, and lung cancer cell lines Wales et al. (1995[32]) named and patented the Hypermethylated In Cancer 1 (HIC1) gene. Since then HIC1 methylation has been confirmed in cell lines and tissues in various cancers. Previous studies have reported of either a deletion or epigenetic silencing of HIC1 in many types of cancers including colorectal cancer (Bagci et al., 2016[3]), breast cancer (Fujii et al., 1998[15]; Wang et al., 2018[33]), and esophageal squamous cell carcinoma (Li et al., 2015[21]).

Numerous studies (Briggs et al., 2008[5]; Van Rechem et al., 2009[31]; Zhang et al., 2010[37]) have reported various possible HIC1 tumor suppressor pathways. However, the most widely accepted among these theories is a model involving the tumor suppressor gene P53. It is generally accepted that HIC1 acts as a tumor suppressor gene through a complex regulation cycle involving HIC1, SIRT1, and P53 (Chen et al., 2005[8]). HIC1 directly interacts with the SIRT1 protein, forming a transcriptional repression complex which binds to and represses the SIRT1 promoter. The P53 tumor suppressor is an important target of SIRT1 which belongs to the type III NAD^+^-dependent histone/protein deacetylases family. However, deacetylation of P53 negatively regulates its activation and thus weakening P53 function such as growth arrest control and apoptosis in response to stress. Further, P53 acts as a positive transcriptional regulator of HIC1. Normally, activated P53 will induce HIC1 expression and in turn repress SIRT1, contributing to the positive feedback. However, in tumor cells, inactivation of HIC1 leads to elevated SIRT1 level, which would deacetylate and inactivate P53 (Jenal et al., 2010[17]).

Earlier studies (Abouzeid et al., 2011[1]; Zhao et al., 2013[38]; Zheng et al., 2013[39]) have suggested the link between the inactivation of HIC1 and HIC1 promoter methylation since promoter methylation is often associated with loss of heterozygosity (LOH) and low expression level of the gene. The promoter hypermethylation has been found in various solid tumors including breast (Fujii et al., 1998[15]) and ovarian cancers (Feng et al., 2008[13]). In addition, hypermethylation of HIC1 promoter is also found in some normal tissues including thyroid (Wu et al., 2016[34]) and colorectal (Pehlivan et al., 2010[24]) tissues. As a result, the specific impact of HIC1 promoter methylation and its principal contribution towards the inactivation of HIC1 in tumors needs further examination. In this study, we explored the specific impact of HIC1 promoter hypermethylation and its association with cancer risk in various solid tumors systematically.

## Materials and Methods

### Data sources and keywords

We searched available studies in PubMed, Google Scholar, Embase, Cochrane Library, and Web of Science databases (last updated search on November 2019). With respect to the keywords applied in our study, we used a combination of subject words and free words (“HIC1” or “HIC ZBTB transcriptional repressor 1” or “Hypermethylated in cancer 1” or “HIC1 protein”) and (“DNA Methylation” [Mesh] or “Methylation” or “Hypermethylation” or “Demethylation”) and (“Neoplasms” [Mesh] or “Cancer” or “Carcinoma” or “tumor”). No other restriction was set to the search, so review articles were also retrieved as the references.

### Selection criteria for eligible studies

Studies that met the following criteria were included in our study: 1. The study investigated the correlation between HIC1 promoter methylation and solid tumors. 2. The study provided sufficient information about the frequency of HIC1 promoter methylation in tissue or other samples of cancer patients. 3. All the studies were independent case-control studies. 4. The total numbers of patients and controls were more than five. 5. An odds ratio (*OR*) with a 95 % confidential interval (*CI*) was reported or could be calculated.

### Literature screening

 Data were extracted independently by two reviewers with a standard extraction table. After an independent thorough search, the titles and abstracts of each available study were judged based on the above-mentioned selection criteria; If the title and abstracts were not representative, we continued to read the full text to check for its suitability. Where there was a disagreement, a third reviewer was asked to review to build consensus.

### Data extraction

The procedure for the retrieval of relevant data from eligible studies was as follows: first author's name, year of publication, sample size, age, gender, ethnicity, disease type, and detection method for HIC1 promoter methylation as well as methylation frequency of HIC1 promoter in cancer samples, normal samples, and benign samples were extracted independently. Any disagreements were resolved through a panel discussion. The characteristics of selected studies used in the meta-analysis are presented in Table 1(Tab. 1) (References in Table 1: Abouzeid et al., 2011[1]; Alvarez et al., 2013[2]; Bagci et al., 2016[3]; Feng et al., 2008[13]; Gustafson et al., 2004[16]; Lenhard et al., 2005[20]; Li et al., 2015[21]; Narayan et al., 2003[22]; Parrella et al., 2005[23]; Pehlivan et al., 2010[24]; Rathi et al., 2003[25]; Uhlmann et al., 2003[30]; Yu et al., 2011[36]; Zhao et al., 2013[38]).

### Statistical analyses

Data were analyzed using STATA 12.0. The odds ratio (*OR*) with 95 % confidential interval (*CI*) was used to estimate the effect side of each study, measuring the risk of HIC1 promoter methylation in cancer versus normal and cancer versus benign. An *OR *> 1 with a 95 % *CI *that does not overlap is an indication of an association of HIC1 promoter methylation with increased cancer risk. The utilization of OR was measured by *Z* test. Heterogeneity was measured by χ^2^ test and* I^2^* test. A fixed-effects model was applied if there was no statistically significant heterogeneity (*P *≥ 0.1, *I^2 ^*< 50 %), while a random-effects model was applied to the meta-analysis when heterogeneity existed among the studies (Chen et al., 2016[7]). Also, subgroup analyses based on sample type, ethnicity, disease type, and detection method were performed to detect methylation and explore the source of heterogeneity. Further, a sensitivity analysis was performed using the live-one-out analysis to check the strength of the effect exerted by individual studies in our meta-analysis. The funnel plot was used to assess the publication bias. *P *value < 0.05 was considered statistically significant.

## Results

### Included studies

**Figure 1:**
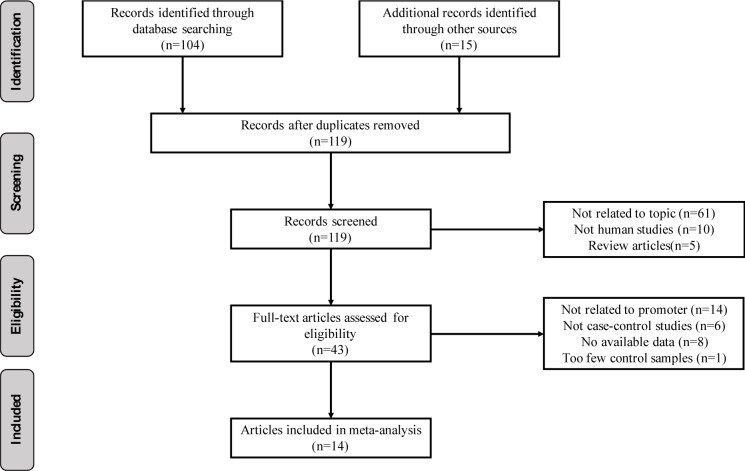
The flow chart of the study selection process

We followed a cautious study selection process as shown in Figure 1(Fig. 1). Forty-three studies were selected after the initial identification and screening processes. After checking the full text, 14 studies were not related to HIC1 promoter methylation. Specifically, 6 studies lacked control samples, 8 studies had some missing data, and the number of control samples in 1 study was less than five. Finally, a total of 14 case-control studies (949 cancer patients, 282 benign, and 371 normal controls) were selected for further analysis. The publication year ranged from 2003 to 2016. The details of the selected studies are summarized in Table 1(Tab. 1). The clinicopathologic informations are shown in supplementary Table 1. 

### Quality control of selected studies was assessed by the Newcastle-Ottawa Scale (NOS)

We used the NOS (Stang, 2010[28]) to access the quality of selected studies and conflicting decisions by the two independent reviewers were resolved in consultation with a third reviewer. Studies with NOS score >5 on the 9-point scoring system were considered to be of high-quality (Table 2(Tab. 2); References in Table 2: Abouzeid et al., 2011[1]; Alvarez et al., 2013[2]; Bagci et al., 2016[3]; Feng et al., 2008[13]; Gustafson et al., 2004[16]; Lenhard et al., 2005[20]; Li et al., 2015[21]; Narayan et al., 2003[22]; Parrella et al., 2005[23]; Pehlivan et al., 2010[24]; Rathi et al., 2003[25]; Uhlmann et al., 2003[30]; Yu et al., 2011[36]; Zhao et al., 2013[38]). 

### Association of HIC1 promoter methylation with solid tumors

**Figure 2:**
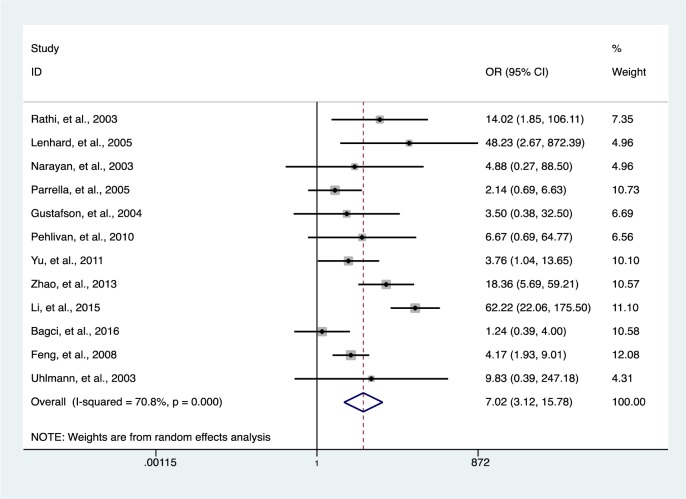
The association between HIC1 promoter methylation and cancer risk using forest plots (cancer versus normal)

**Figure 3:**
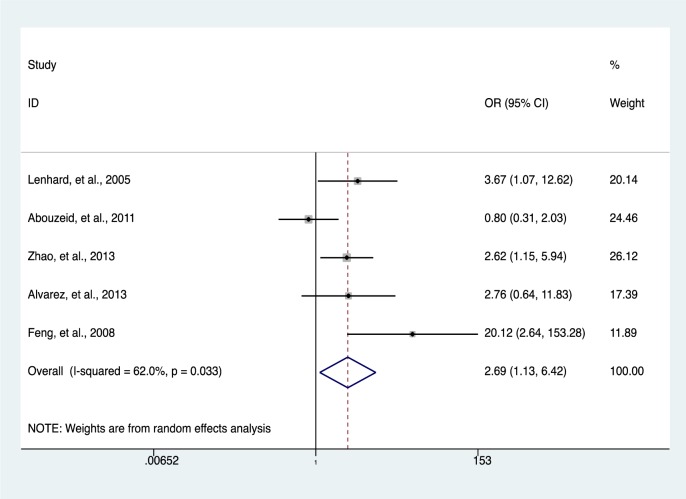
The association between HIC1 promoter methylation and cancer risk using forest plots (cancer versus benign)

**Figure 4:**
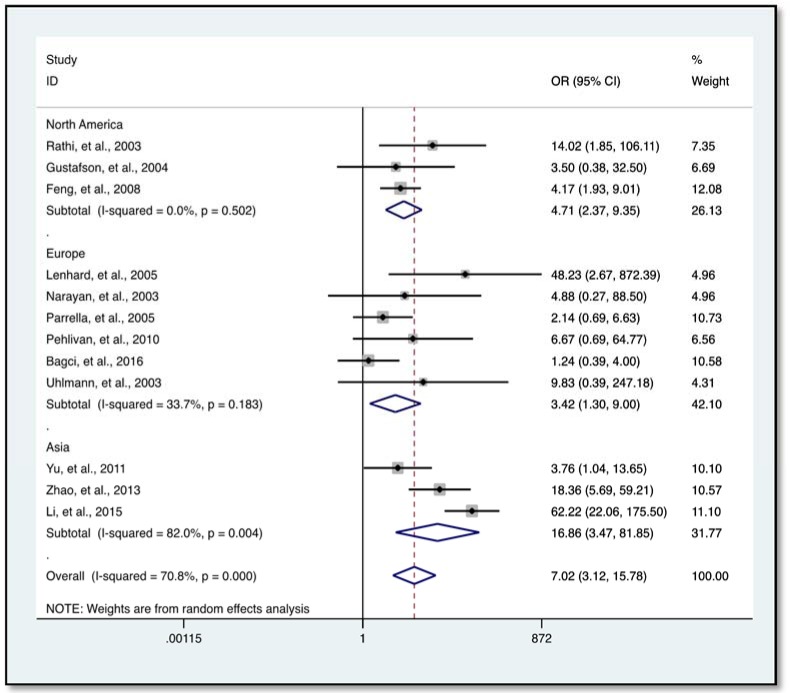
Subgroup analysis stratified by ethnicity using forest plot

Our data indicates a significantly elevated HIC1 promoter methylation in tumor samples compared to normal (*OR* = 7.02, 95 % *CI* 3.12-15.78, *P* < 0.001) (Figure 2(Fig. 2); References in Figure 2: Bagci et al., 2016[3]; Feng et al., 2008[13]; Gustafson et al., 2004[16]; Lenhard et al., 2005[20]; Li et al., 2015[21]; Narayan et al., 2003[22]; Parrella et al., 2005[23]; Pehlivan et al., 2010[24]; Rathi et al., 2003[25]; Uhlmann et al., 2003[30]; Yu et al., 2011[36]; Zhao et al., 2013[38]) and benign controls (*OR* = 2.69, 95 % *CI *1.13-6.42, *P* = 0.025) (Figure 3(Fig. 3); References in Figure 3: Abouzeid et al., 2011[1]; Alvarez et al., 2013[2]; Feng et al., 2008[13]; Lenhard et al., 2005[20]; Zhao et al., 2013[38]). However, the data showed a significant level of heterogeneity (*I^2^* = 70.8 %, *P *< 0.001), hence the random-effects model was used for the meta-analysis (Chen et al., 2016[7]; Dou et al., 2018[11]). To explore the source of heterogeneity, subgroup analyses were conducted by stratifying data according to ethnicity, cancer type, methylation detection method and sample type. Subgroup analysis based on ethnicity revealed an insignificant level of heterogeneity among North Americans (*I^2^* = 0.0 %, *P *= 0.502) and Europeans (*I^2^* = 33.7 %, *P *= 0.183). In contrast, there was no reduction in heterogeneity among Asian samples even though the level of HIC1 methylation of cancer samples among these group was higher (*OR* = 16.86, 95 % *CI* 3.47-81.85, *P *< 0.001) compared to that among North America (*OR* = 4.71, 95 % *CI* 2.37-9.35, *P *< 0.001) and Europe (*OR* = 3.42, 95 % *CI* 1.30-9.00, *P *= 0.013) (Figure 4(Fig. 4); References in Figure 4: Bagci et al., 2016[3]; Feng et al., 2008[13]; Gustafson et al., 2004[16]; Lenhard et al., 2005[20]; Li et al., 2015[21]; Narayan et al., 2003[22]; Parrella et al., 2005[23]; Pehlivan et al., 2010[24]; Rathi et al., 2003[25]; Uhlmann et al., 2003[30]; Yu et al., 2011[36]; Zhao et al., 2013[38]). 

**Figure 5:**
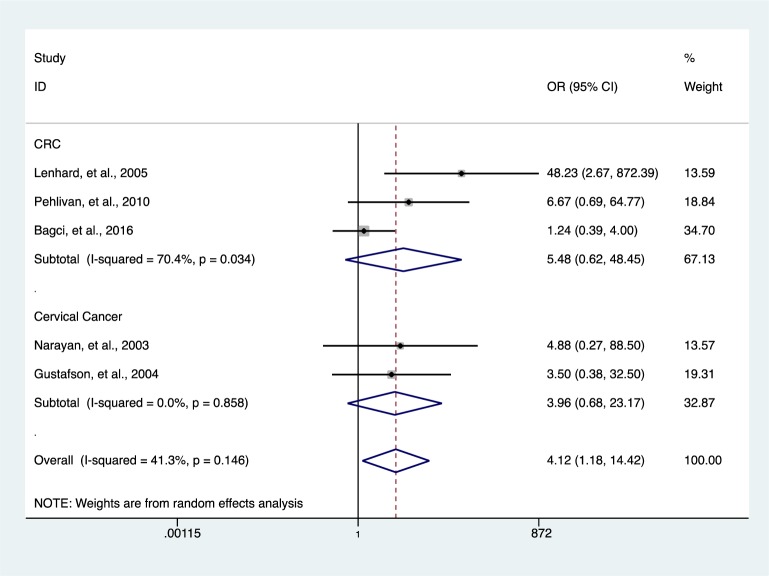
Subgroup analysis according to cancer type using forest plot

**Figure 6:**
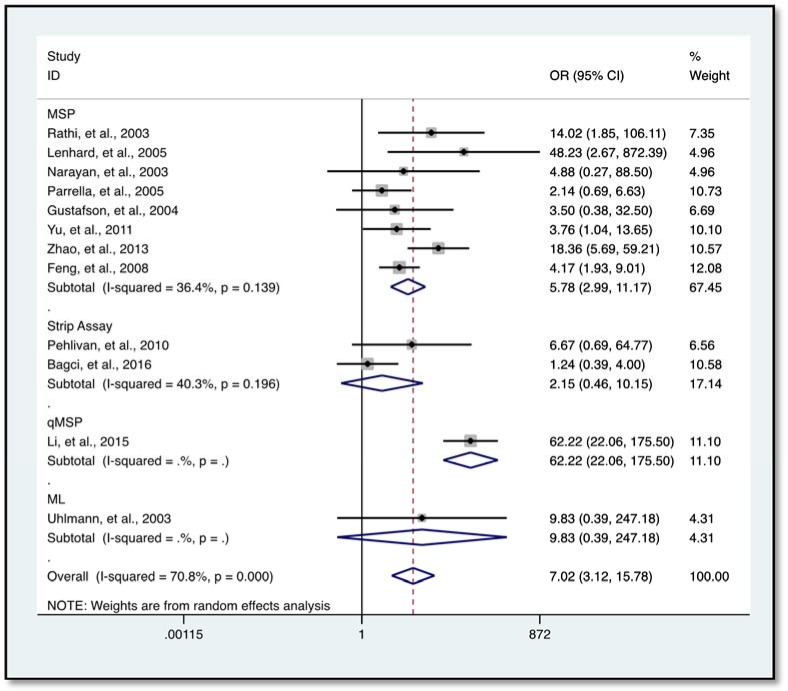
Subgroup analysis according to methylation detection method using forest plot

**Figure 7:**
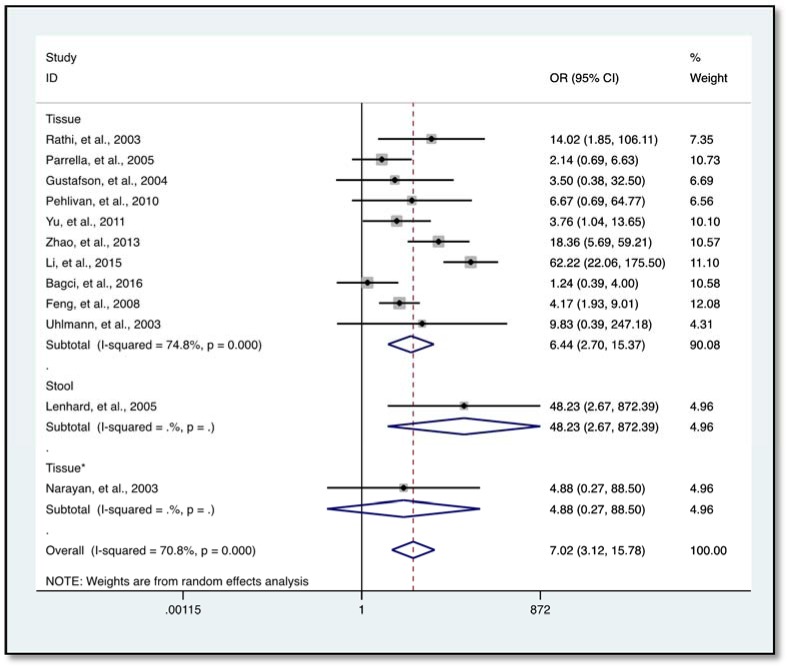
Subgroup analysis according to sample type using forest plot

Further analysis showed a reduced level of heterogeneity in cervical cancer (*I^2 ^*= 0.0 %, *P *= 0.858) and high heterogeneity in colorectal cancer (CRC) (*I^2^* = 70.4 %, *P *= 0.034). However, stratified analysis based on cervical cancer (*OR* = 3.96, 95 % *CI* 0.68-23.17, *P *= 0.127) and CRC (*OR* = 5.48, 95 % *CI* 0.62-48.45, *P *= 0.126) did not reveal a significantly elevated HIC1 promoter methylation (Figure 5(Fig. 5); References in Figure 5: Bagci et al., 2016[3]; Gustafson et al., 2004[16]; Lenhard et al., 2005[20]; Narayan et al., 2003[22]; Pehlivan et al., 2010[24]). Categorization based on methylation detection method showed that HIC1 promoter methylation was significantly associated with cancer risk (*OR* = 5.78, 95 % *CI* 2.99-11.17, *P *< 0.001) with a significant reduction in heterogeneity (*I^2^* = 36.4 %, *P *= 0.139) among samples that employed methylation specific PCR(MSP) detection method (Figure 6(Fig. 6); References in Figure 6: Bagci et al., 2016[3]; Feng et al., 2008[13]; Gustafson et al., 2004[16]; Lenhard et al., 2005[20]; Li et al., 2015[21]; Narayan et al., 2003[22]; Parrella et al., 2005[23]; Pehlivan et al., 2010[24]; Rathi et al., 2003[25]; Uhlmann et al., 2003[30]; Yu et al., 2011[36]; Zhao et al., 2013[38]). Heterogeneity was however high among various cancer tissues samples (*I^2^* = 74.8 %, *P *= 0.000) (Figure 7(Fig. 7); References in Figure 7: Abouzeid et al., 2011[1]; Alvarez et al., 2013[2]; Bagci et al., 2016[3]; Feng et al., 2008[13]; Gustafson et al., 2004[16]; Lenhard et al., 2005[20]; Li et al., 2015[21]; Narayan et al., 2003[22]; Parrella et al., 2005[23]; Pehlivan et al., 2010[24]; Rathi et al., 2003[25]; Uhlmann et al., 2003[30]; Yu et al., 2011[36]; Zhao et al., 2013[38]).

### Analysis of sensitivity and publication bias

**Figure 8:**
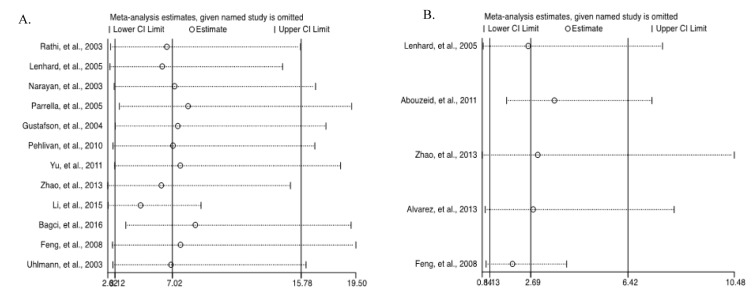
Sensitivity analysis of the summary odds ratio coefficients on the associations between HIC1 promoter methylation and the pathogenesis of human tumors. (A) Cancer versus normal; (B) Cancer versus benign

**Figure 9:**
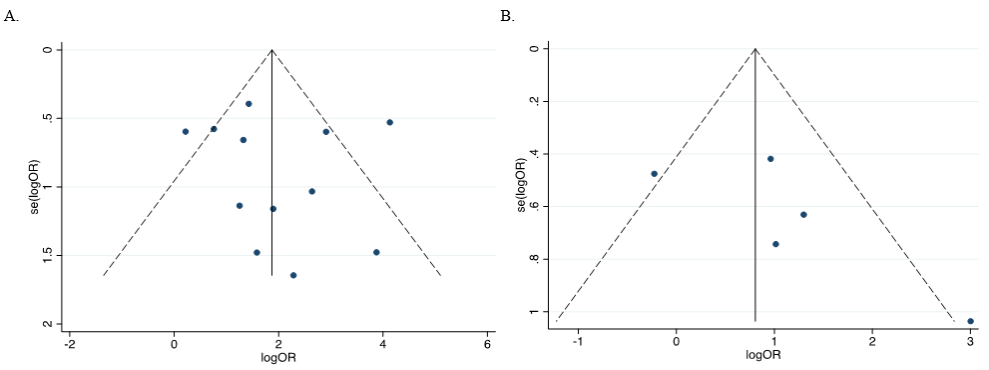
Assessment of publication bias in the evaluation of HIC1 promoter methylation and cancer risk using a funnel plot. (A) Cancer versus normal (Egger's test: *t*=0.35, *p*=0.735); (B) Cancer versus benign (Egger's test: *t*=1.57, *p*=0.241)

The leave-one-out sensitivity analysis revealed that no single study significantly influenced the overall (Figure 8A and 8B(Fig. 8); References in Figure 8: Bagci et al., 2016[3]; Feng et al., 2008[13]; Gustafson et al., 2004[16]; Lenhard et al., 2005[20]; Li et al., 2015[21]; Narayan et al., 2003[22]; Parrella et al., 2005[23]; Pehlivan et al., 2010[24]; Rathi et al., 2003[25]; Uhlmann et al., 2003[30]; Yu et al., 2011[36]; Zhao et al., 2013[38]) outcome. In addition, funnel plots revealed no potential publication bias of the selected studies for both cancer versus normal (Egger's test: *t*=0.35, *p*=0.735) (Figures 9A(Fig. 9)) and cancer versus benign (Egger's test: *t*=1.57, *p*=0.241) (Figure 9B(Fig. 9)).

## Discussion

Cancer has been described for a long time as a genetically driven modified cluster of diseases catalyzed by modifications involving chromosomes, oncogenes and tumor suppressor genes. However, recent findings point to the increasing involvement of epigenetic changes expressed through DNA methylation and histone tail modifications, which demonstrates the importance of heritable gene expression patterns as a focal point of many human diseases including cancer (Fleuriel et al., 2009[14]). Although cancer is becoming an international burden in both advanced and less developed countries, early diagnosis still remains a problem. DNA methylation can be a potential hallmark for cancer (Jones and Baylin, 2007[18]). In line with this concept, the potential of HIC1 promoter methylation to act as biomarkers has been assessed in several types of cancers (Chen et al., 2017[6]; Yu et al., 2011[36]; Zheng et al., 2013[39]), albeit with some inconclusiveness in their outcomes. We sought to perform a more accurate and comprehensive meta-analysis to assess the association between HIC1 promoter methylation and cancer risk. Our findings revealed a significantly elevated HIC1 promoter methylation in tumor compared to the healthy and benign controls. Even though the level of overall heterogeneity in our selected samples (different cancer sub-types and different sample types) seems to be high (I^2^=70.8 % and 62.0 % respectively for cancer vs healthy controls and cancer vs benign controls), it is highly comparable to that reported in a previous study (I^2^=71.8 %) which analyzed tissue samples from a single cancer sub-type (Dou et al., 2018[11]). We also report of a relatively reduced level of heterogenicity among Europeans and North American subgroups. In addition, methylation specific PCR (MSP) was the most reliable detection method which showed a low level of heterogeneity among studies. 

In line with our findings, Yu et al. (2011[36]) reported a significantly high HIC1 promoter methylation level in gastric cancer compared to the control. Hence confirming the relationship between HIC1 promoter methylation and cancer risk. Tumorigenesis has been shown to be characterized by the hypermethylation of cytosines 5′ to guanosines (CpG) occurring in the promoter region in the genomic DNA of tumor suppressor genes (Baylln et al., 1998[4]; Rush et al., 2001[26]; Smiraglia and Plass, 2002[27]). Due to the fact 5-methylcytosine is usually not stable it has the potential of mutating to thymine thus causing the degradation of methylated CpG to TpG/CpA (Yang and Park, 2012[35]). In most tumors, the aberrant hypermethylation of CpG islands results in the silencing of suppressor genes such as HIC1 leading to the exacerbation of the carcinogenesis.

Contrary to our overall outcome, Alvarez et al. in a study that employed gastric cancer patients and chronic gastritis patients as control reported that among three genes (THBS1, GATA-4, and HIC1), HIC1 was least methylated. They further ascertain that HIC1 methylation may not be the principal mechanism implicated in its down-regulation in gastric cancer samples. However, their results showed an increasing trend of methylation from pre-cancerous tissues to cancerous tissues but were not significant (Alvarez et al., 2013[2]).

Interestingly, samples analyzed using MSP (known for its simple design, execution and high sensitivity in the ability to detect small quantities of methylated DNA (Derks et al., 2004[10]; Fackler et al., 2004[12]) revealed a significant association* (OR* = 5.78, 95 % *CI* 2.99-11.17, *P* < 0.001) of HIC1 promoter methylation with cancer risk. This was observed irrespective of ethnicity, cancer type, or sample type with a subsequent reduction in the level of heterogeneity (*I^2 ^*= 36.4 %, *P *= 0.139) among the samples. This sensitivity and specificity of MSP in detecting promoter methylation corroborates findings from an earlier study which also reported a relatively lower level of heterogeneity among prostate cancer samples when MSP was used (Dou et al., 2018[11]). Also, HIC1 promoter methylation detected with the reverse-hybridization strip assay (RSA) (*I^2 ^*= 40.3 %, *P *= 0.196) showed a reduced level of heterogeneity, however, there was no significant association of hypermethylation with cancer risk among the samples. From the above dynamics, it is possible to infer that the high level of heterogeneity in the overall outcome could possibly be a result of the differences in the methylation detection methods. It is therefore important that methylation detection methods are standardized to achieve consensus. 

Stratifications based on ethnicity did not show any significant heterogeneity among European and North American samples. However samples from Asia, specifically China, had increased level of heterogenicity. It is, however, informative to note the differences in the methylation detection methods employed which could be a possible cause of this heterogenicity. Also, the complexities and wide variation in the environmental and genetic background, as well as sample size, could contribute to such heterogeneities (Dou et al., 2018[11]). Sub-group analysis based on cancer type was not possible in most cases because most cancer sub-types contained only one study except that heterogeneity was low in cervical cancer (*I^2 ^*= 0.0 %, *P *= 0.858) and high in CRC (*I^2 ^*= 70.4 %, *P *= 0.034).

Even though our data conclusively point to HIC1 promoter methylation associated cancer risk, we acknowledge the absence of some relevant socio-demographic data such as age and gender for further analysis. Also, our findings may be limited by factors such as relatively small samples' size in some selected studies. We also acknowledge the possibility of publication bias probably due to the selection criteria of the studies which made provisions for only published studies communicated in English language. Finally, it seems that DNA hypermethylation is not a random process and could accurately characterize type, stage or histology of specific tumors (Kulis and Esteller, 2010[19]). However, our analysis is limited by lack of data in this regard hence preventing a deeper probe into the correlation of HIC1 promoter methylation with cancer subtype and stage. 

## Conclusion

In summary, our results indicate that HIC1 promoter methylation may contribute to cancer risk in several cancer samples, suggestive of the close connection of HIC1 promoter methylation with cancer development. It is also important that methylation detection methods are stream lined to achieve a consensus as MSP was observed to be the most sensitive and specific method for detection among our selected studies.

## Conflict of interest

The authors declare no conflict of interest.

## Authors’ contribution

JY conceived and edited the article, TZ, JA, and DW wrote the article.

## Acknowledgements

This work was supported by the Program for Changjiang Scholars and Innovative Research Team in University of China (IRT1230).

## Supplementary Material

Supplementary information

## Figures and Tables

**Table 1:**
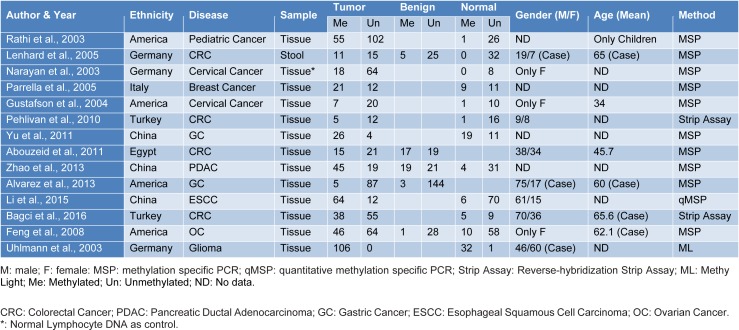
Characteristics of selected studies in the meta-analysis

**Table 2:**
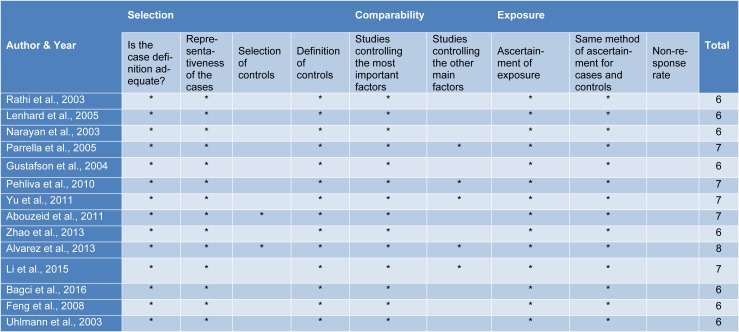
The Newcastle-Ottawa Scale (NOS) for assessing the quality of case-control studies
