# Supplementary material for: Association between HIC1 promoter methylation and solid tumor: A meta-analysis
Source: EXCLI J. 2020 Apr 7;19:476–89. doi: 10.17179/excli2020-1102 (PMC7214777; doi:10.17179/excli2020-1102)
Supplement: Supplementary information [file EXCLI-19-476-s-001.pdf]

EXCLI Journal 2020;19:476-489 – ISSN 1611-2156
Received: February 03, 2020, accepted: March 27, 2020, published: April 07, 2020

Supplementary information to:

ASSOCIATION BETWEEN HIC1 PROMOTER METHYLATION AND SOLID TUMOR: A META-ANALYSIS

Tie Zhao[1], Justice Afrifa[1,2], Dong Wang[1], Jingcui Yu[1*]

[1] Scientific Research Centre, The Second Affiliated Hospital of Harbin Medical University, Harbin 150081, China
[2] Department of Medical Laboratory Science, University of Cape Coast, Cape Coast, Ghana

[*] **Corresponding author:** Jingcui Yu (MD, Ph.D.), Scientific Research Centre, The Second Affiliated Hospital of Harbin Medical University, 246 Xuefu Road, Nangang District, Harbin 150081, China; phone: +86-451-86605908; E-mail: yujingcui@ems.hrbmu.edu.cn

http://dx.doi.org/10.17179/excli2020-1102

This is an Open Access article distributed under the terms of the Creative Commons Attribution License (http://creativecommons.org/licenses/by/4.0/).

EXCLI Journal 2020;19:476-489 – ISSN 1611-2156
Received: February 03, 2020, accepted: March 27, 2020, published: April 07, 2020

Supplementary Table 1: Clinicopathologic data of selected studies in meta-analysis

Author & Year	Disease	Localization	Diameter	Histological differentiation	Stage	Family history
Rathi et al., 2003	Pediatric Cancer	ND	ND	ND	ND	ND
Lenhard et al., 2005	CRC	ND	ND	ND	I/II: 6<br>III/IV: 20	ND
Narayan et al., 2003	Cervical Cancer	ND	<6cm: 32<br>≥6cm: 38	ND	I/II: 36<br>III/IV: 46	ND
Parrella et al., 2005	Breast Cancer	ND	ND	ND	ND	ND
Gustafson et al., 2004	Cervical Cancer	ND	ND	ND	ND	ND
Pehlivan et al., 2010	CRC	ND	ND	ND	ND	ND
Yu et al., 2011	GC	ND	ND	ND	ND	ND
Abouzeid et al., 2011	CRC	Right: 5<br>Left: 25<br>Unknown: 6	ND	ND	I/II: 24<br>III/IV: 12	No: 29<br>Yes: 7
Zhao et al., 2013	PDAC	ND	<6cm: 39<br>≥6cm: 25	High-Mid: 18<br>Low: 46	I/II: 33<br>III/IV: 31	ND
Alvarez et al., 2013	GC	ND	ND	ND	ND	ND
Li et al., 2015	ESCC	Upper-Mid: 46<br>Lower: 30	<5cm: 36<br>≥5cm: 40	High-Mid: 62<br>Low: 15	I/II: 35<br>III/IV: 41	No: 60<br>Yes: 16
Bagci et al., 2016	CRC	ND	ND	ND	ND	ND
Feng et al., 2008	OC	ND	ND	ND	I/II: 21<br>III/IV: 79<br>LMP: 10	ND
Uhlmann et al., 2003	Glioma	ND	ND	ND	ND	ND

CRC: Colorectal Cancer; PDAC: Pancreatic Ductal Adenocarcinoma; GC: Gastric Cancer; ESCC: Esophageal Squamous Cell Carcinoma; OC: Ovarian Cancer; LMP: Neoplasia Low Malignant Potential; ND: no data.
